# Active disease and its associated factors among patients with inflammatory bowel disease in Addis Ababa, Ethiopia: a hospital-based cross-sectional study

**DOI:** 10.3389/fgstr.2025.1569933

**Published:** 2025-08-28

**Authors:** Zinabu Desalegn, Abate Bane, Guda Merdassa, Amanuel Arota

**Affiliations:** ^1^ Department of Internal Medicine, School of Medicine, Wolaita Sodo University, Wolaita Sodo, Ethiopia; ^2^ Division of Gastroenterology, Department of Internal Medicine, College of Health Sciences, Addis Ababa University, Addis Ababa, Ethiopia; ^3^ Department of Epidemiology and Biostatistics, School of Public Health, Wolaita Sodo University, Wolaita Sodo, Ethiopia

**Keywords:** inflammatory bowel disease, nutritional screening tools, nutrition, factors, Ethiopia

## Abstract

**Introduction:**

Inflammatory bowel disease (IBD) is a chronic inflammatory condition affecting the gastrointestinal tract, primarily classified into Crohn’s disease (CD) and ulcerative colitis (UC), with some cases falling into the indeterminate or unclassified category. A significant number of individuals with IBD may present with active disease, which contributes substantially to complications. Therefore, early detection of patients with clinically active disease is essential for timely referral and appropriate management to prevent related complications. This study aimed to assess the prevalence of active disease and its associated factors among patients with IBD.

**Methods:**

A hospital-based cross-sectional study was conducted at Tikur Anbessa Specialized Hospital and Adera Medical and Surgical Center, Addis Ababa, Ethiopia, in 2024. A total of 252 patients with IBD were selected using a consecutive recruitment technique. Data were collected from medical records and patient interviews using a structured questionnaire. Bivariate logistic regression was performed, followed by multivariable analysis to examine the association between the outcome and predictor variables. Variables with a p-value ≤ 0.25 in the bivariate analysis were included in the multivariable model. A p-value < 0.05 was considered statistically significant.

**Result:**

A total of 242 individuals participated in the study, yielding a response rate of 96.03%. More than one-third, 82 (33.88%), of the patients had active disease at the time of inclusion. The majority of IBD patients, 190 (78.51%), were diagnosed with CD. Patients with a monthly income between 500–1000 Ethiopian Birr (ETB) had an approximately 80% lower risk of active disease compared to those with an income of less than 500 ETB (AOR = 0.20; 95% CI: 0.05–0.79). Patients identified as being at high risk for malnutrition based on the Malnutrition Universal Screening Tool (MUST) score had about four times higher risk of active disease compared to those at low risk (AOR = 4.30; 95% CI: 1.69–10.91).

**Conclusion:**

One in every three IBD patients had active disease. MUST score and income level were found to be significant predictors of disease activity. Targeted interventions addressing nutritional, clinical, and socioeconomic determinants of IBD outcomes should be implemented.

## Introduction

Inflammatory bowel disease (IBD) is a chronic inflammatory condition of the gastrointestinal tract, classified into Crohn’s disease, ulcerative colitis, and indeterminate colitis (1). These conditions are characterized by chronic inflammation of the intestinal mucosa, resulting from complex interactions among genetic, environmental, immunologic, and intestinal microbial factors (2). The incidence of IBD is increasing globally, including in Africa (3–5). The shift from an agriculture-based lifestyle to an industrial and post-industrial mode, along with changes from fiber-rich diets to industrial fast food, is believed to be among the environmental factors contributing to the growing burden of IBD worldwide (6, 7).

IBD is associated with a wide range of complications that may affect both the gastrointestinal tract and other organ systems (8, 9). Clinical disease activity plays a central role in the development and severity of these complications, particularly malnutrition (10). Disease activity refers to the presence and severity of inflammation in the gastrointestinal tract, which correlates with clinical symptoms and pathological changes and reflects the dynamic nature of the disease, fluctuating between periods of active inflammation (flare-ups) and remission (10, 11). Assessing clinical activity in IBD is crucial for monitoring disease progression and adjusting treatment strategies (12). While individual evaluations often rely on subjective symptoms, clinical trials require objective and reproducible indices (13). Currently, several disease activity indices exist for both Crohn’s disease and ulcerative colitis, but no single scoring system is applicable to both conditions (10, 14) due to variations in study objectives and patient characteristics. Despite extensive research, no definitive method has been established to assess disease activity in IBD, as all clinical indices involve a high degree of subjectivity and are subject to considerable interobserver variation—even among experienced researchers (15). In everyday clinical practice, most gastroenterologists rely on global clinical judgment, which, while less reproducible, is simpler and more feasible for decision-making (16). Parameters commonly used to assess disease activity in IBD include laboratory markers such as erythrocyte sedimentation rate (ESR), albumin, hemoglobin (Hgb), hematocrit, platelet count, C-reactive protein (CRP), fibronectin, human leukocyte elastase, and lymphocyte T9-receptor involvement (17, 18). In addition to these biomarkers, clinical activity indices such as the Mayo Score for ulcerative colitis and the Harvey–Bradshaw Index (HBI) for Crohn’s disease are widely used to assess disease severity by combining clinical symptoms with laboratory data (10, 17, 19).

The burden of active disease at inclusion among IBD patients ranges from 22% to 44% (20, 21). Moreover, malnutrition is more common among IBD patients with prevalence rates ranging from 6.1% to 69.7%, depending on disease type, activity, and assessment methods (9, 22–24). Malnutrition in IBD results from a complex interplay of factors, including chronic inflammation that increases metabolic demands and impairs nutrient absorption, dietary restrictions, and gastrointestinal symptoms (25–28). The intricate interplay between the chronic inflammatory nature of IBD, and the potential effect of IBD on nutrient absorption raises concerns about the potential nutritional risk faced by these patients (29). Nutrition screening identifies patients with or at risk of under nutrition who will subsequently be referred for comprehensive dietetic assessment (30). Despite advancements in medical interventions and therapeutic strategies for managing IBD, the impact of nutritional status on the clinical outcomes of patients’ like clinical activity of disease remains inadequately understood (31).

Globally, studies have shown that higher disease activity correlates with malnutrition, leading to more frequent hospitalizations and surgical interventions (32). In high-income countries, routine nutritional screening is implemented, enabling healthcare providers to identify at-risk patients and tailor interventions that mitigate complications (33). Evaluation of nutritional status at admission, particularly in active disease is essential because early medical nutrition therapy can decrease disease morbidity and improve quality of life (31). Given the increased prevalence of nutrient deficiencies and the influence of socioeconomic factors in low-income settings, integrating nutritional assessment into standard IBD care is significant to optimize patient health and treatment success (34). However, in many African countries, including Ethiopia, such systematic approaches to nutritional screening are lacking, leading to delayed interventions and worsened health outcomes. There is no adequate data on the assessment of clinical disease activity and its association with malnutrition and other factors among IBD patients in Ethiopia. Thus, the aim of this study was to assess the prevalence of active disease at inclusion and its association with nutritional status and other factors among patients with inflammatory bowel disease at Tikur Anbessa Specialized Hospital and Adera Medical and Surgical Center, Addis Ababa, Ethiopia, in 2024. The information obtained adds important locally applicable knowledge and improves awareness of the relevance of nutritional evaluation and its impact on patient outcomes in clinical practice in our country and similar low-income settings.

## Methods

### Study area, study design, and study period

A hospital-based prospective cross-sectional study was conducted at Adera Medical and Surgical Center (AMSC) and Tikur Anbessa Specialized Hospital (TASH), both located in Addis Ababa, the capital city of Ethiopia. These hospitals have gastroenterology and hepatology divisions that provide training for medical residents, gastroenterology fellows, and undergraduate students, alongside various clinical services. The units offer diagnostic and therapeutic endoscopy services, as well as inpatient and outpatient care. The study was conducted from February to July 2024.

Population: The source population consisted of IBD patients diagnosed with Crohn’s disease (CD) or ulcerative colitis (UC) who were receiving follow-up care in the outpatient departments of TASH and AMSC. The study population included IBD patients diagnosed with CD or UC who attended follow-up visits in the outpatient departments of the selected hospitals during the study period.

Eligibility: All adult IBD patients aged 18 years and above, diagnosed with CD or UC and receiving follow-up care during the study period, were included in the study. Patients who were severely ill, pregnant, had incomplete medical charts, or were unable to communicate were excluded.

### Sample size determination and sampling procedure

The sample size was calculated using a single population proportion formula with the following parameters: significance level (α) = 95% and maximum acceptable difference (absolute precision) (d) = 0.05. A prevalence rate of 82.8% was taken from a previous study conducted among IBD patients in a North Indian cohort with CD (35). The calculated sample size was 219. After adding a 15% non-response rate, the final sample size became 252. A consecutive sampling technique was used to select the study participants.

### Study variables

Dependent variables: The presence or absence of active disease at inclusion among IBD patients.

Independent variables: The socio-demographic factors (sex, age, education status, residence, marital status, occupation), clinical factors, and nutritional factors.

### Data collection procedures and data collection tool

Data were collected from both medical records and patient interviews using a structured questionnaire, after obtaining verbal informed consent from the participants. The questionnaire and checklist were developed by reviewing various relevant literature sources. Data on sociodemographic characteristics were collected, and anthropometric measurements were recorded.

To collect body mass index (BMI) data, each participant’s weight (in kilograms) and height (in meters) were measured accurately, ensuring that participants were not wearing shoes or heavy clothing. BMI was then calculated by dividing weight by the square of height (BMI = weight/height²). To collect mid-upper arm circumference (MUAC) data, the midpoint between the shoulder and elbow on the participant’s left arm was identified. A MUAC tape was used to measure the circumference at this point, ensuring that the arm was relaxed and hanging by the side. The measurement was recorded in centimeters without compressing the arm.

Clinical activity was assessed using the Partial Mayo Score for ulcerative colitis (UC) and the Harvey–Bradshaw Index (HBI) for Crohn’s disease (CD). Information on treatment at the time of inclusion, as well as laboratory values including hemoglobin (Hgb), C-reactive protein (CRP), erythrocyte sedimentation rate (ESR), and leukocyte count, was collected. Additional information—such as age at diagnosis, extent of UC, location and behavior of CD, and prior hospital admissions or surgeries up to the time of the current visit—was extracted from the medical records.

### Data quality assurance

The questionnaire was initially prepared in English and then translated into Amharic. To ensure consistency, the Amharic version was back-translated into English. Data were collected by general practitioners who received training from the principal investigator. The questionnaire was pretested on 5% of the total sample size one week prior to the actual data collection. Additionally, the principal investigator provided daily feedback and corrections to the data collectors. The completeness, accuracy, consistency, and clarity of the collected data were regularly checked.

### Data management and analysis

The collected data were entered into EpiData version 4.6.0.2 and analyzed using SPSS version 25. Frequencies and corresponding percentages were used to summarize categorical variables, while means with standard deviations and medians with interquartile ranges were used to summarize continuous variables. Continuous variables were compared using Student’s t-test, and categorical variables were compared using the chi-squared test based on clinical disease activity. Results were presented in tables and graphs according to the type of variable.

Bivariate logistic regression analysis was conducted to assess the association between explanatory variables and the outcome variable. Variables with a p-value ≤0.25 in the bivariate analysis were included in the multivariable logistic regression model. Odds ratios (ORs) with 95% confidence intervals (CIs) were used to measure the strength of association between the dependent and independent variables. A p-value <0.05 was considered statistically significant. The goodness of fit of the final model was assessed using the Hosmer–Lemeshow test. Multicollinearity was evaluated using tolerance values and the variance inflation factor (VIF).

### Operational definitions

Malnutrition: A patient was considered to be malnourished if he/she had one of the following: a BMI below 18.5 kg/m^2^, or an Subjective Global Assessment (SGA) grade B or C (36).

Body mass index: Classified as follows: underweight: <18.5 kg/m^2^; normal: 18.5–25 kg/m^2^; overweight >25–29.9 kg/m^2^; obesity 30 kg/m^2^ (36).

Malnutrition Universal Screening Tool (MUST): MUST scores were classified as follows: 0= Low risk for malnutrition, 1= Medium risk for malnutrition, ≥ 2= High risk for malnutrition, refer to nutritional support team (37).

Subjective Global Assessment: SGA was classified as: grade A, well nourished; grade B, moderately malnourished; and grade C, severely malnourished (38).

Disease activity: For luminal CD, clinical activity was defined as a HBI score >4 points (39). For UC, clinical activity was defined as a partial Mayo score >2 points (19).

MUAC category: < 18.0 cm (Adults includes both non-pregnant, pregnant, and postpartum adults) categorized as severe, 8–21 cm categorized moderate and > 21 cm were categorized normal (40).

## Results

### Sociodemographic characteristics

**Figure 1:**
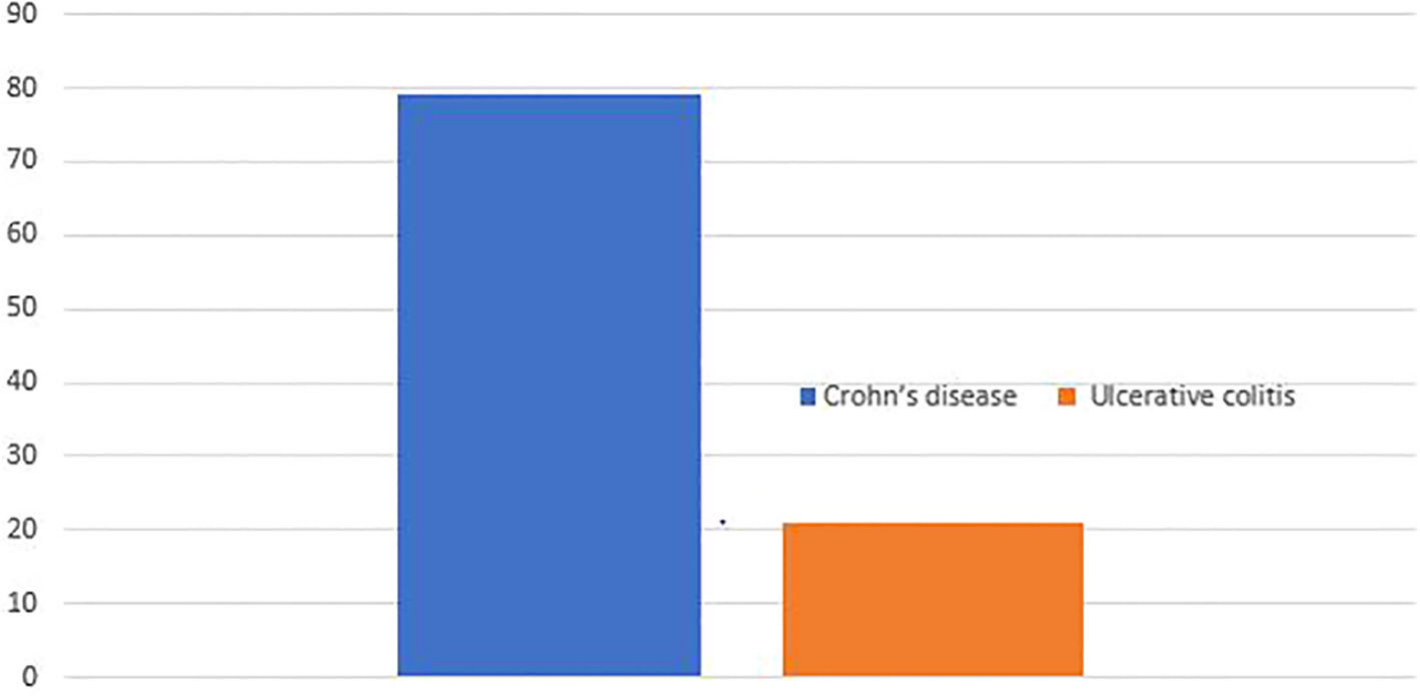
Types of inflammatory bowel disease Addis Ababa Ethiopia, 2024.

A total of 242 individuals participated in the study, yielding a response rate of 96.03%. The majority, 190 (78.51%), had Crohn’s disease (CD), while the remaining 52 (21.49%) had ulcerative colitis (UC) (**Figure 1**).

**Figure 2:**
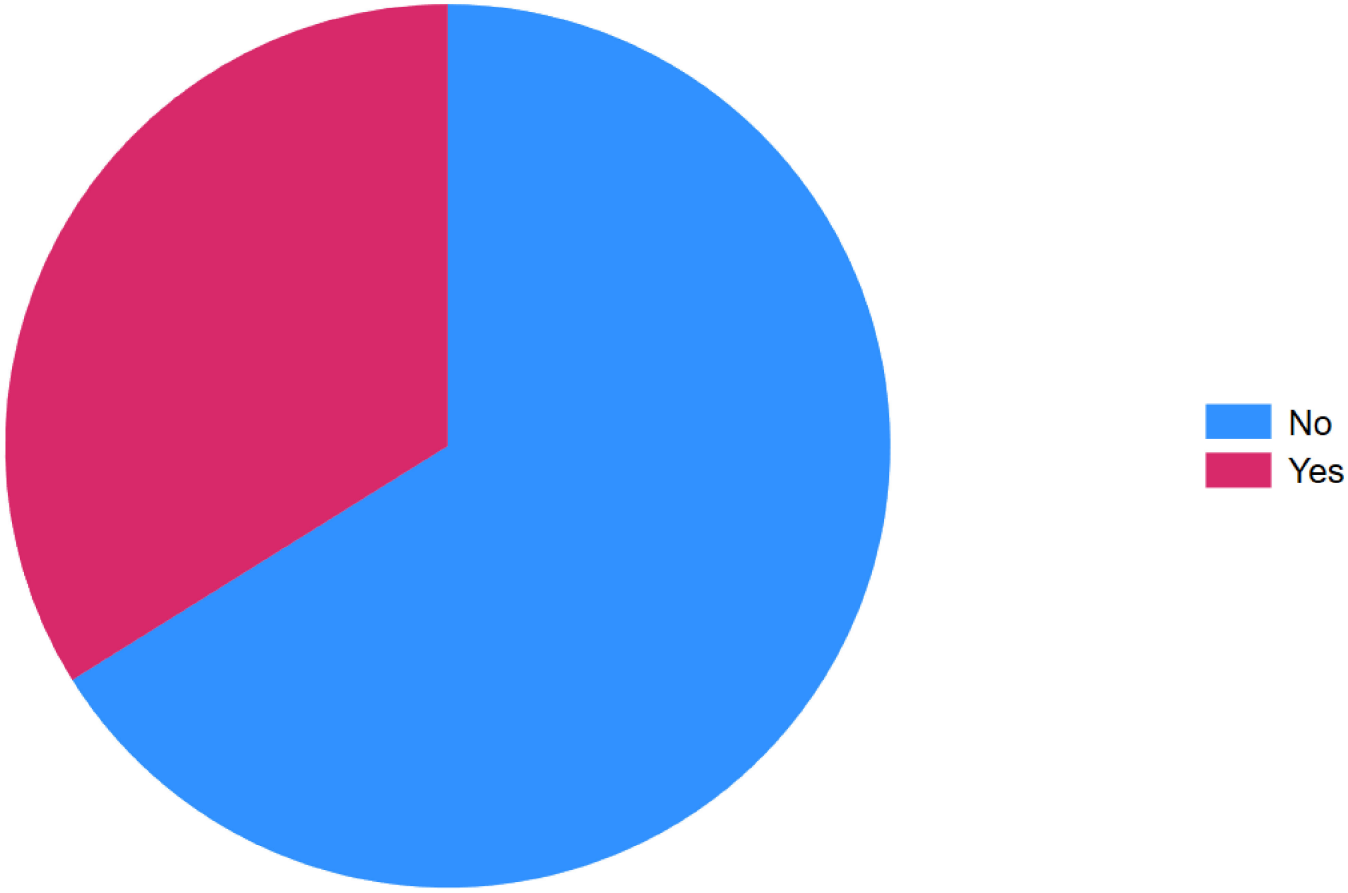
Clinical activity at inclusion of inflammatory bowel disease, Addis Ababa Ethiopia, 2024.

Overall, 160 (66.12%) patients showed no clinical features of active IBD, whereas 82 (33.88%) exhibited features of active disease at the time of inclusion (**Figure 2**). The majority of participants were female, accounting for 159 (65.7%). The mean age was 32.27 years (standard deviation [SD] = 10 years). Regarding marital status, 48.35% were married, 46.49% were single, and smaller proportions were widowed (1.65%) or divorced (3.31%).

Most participants, 228 (94.21%), resided in urban areas. Educational attainment varied: 46.28% had a college education or higher, 33.48% had completed grades 9–12, 12.4% had education between grades 1–8, and 7.43% had no formal education. Employment status showed that 36.36% were employed and 32.64% were currently unemployed. Income data revealed that 59.17% of participants earned between 1,001 and 5,000 Ethiopian Birr (ETB) (**Table 1**).

**Table 1:** Demographic and socioeconomic profile of IBD patients in Addis Ababa, Ethiopia, 2024.

No	Variable	Category	Total (%)	CD (190)		UC (52)		P value
				Active	Non active	Active	Non active	
1	Sex	Male Female	83 (34.30) 159 (65.70)	23 37	40 90	11 11	9 21	0.093
2	Age (year)	Mean ±SD	32.27 ± 10.77	31.52 ± 10.28		35±12.09		0.802
3	Marital status	Single Married Divorced Widowed	113 (46.49) 117 (48.35) 8 (3.31) 4 (1.65)	33 21 5 1	63 63 3 1	6 15 0 1	11 18 0 1	0.070
4	Residence	Urban Rural	228 (94.21) 14 (5.79)	57 3	125 5	18 4	28 2	0.189
5	Level of education	No formal education Grade 1-8 Grade 9-12 College and above	18 (7.44) 30 (12.40) 82 (33.88) 112 (46.28)	4 10 19 27	9 13 45 63	3 3 6 10	2 4 12 12	0.603
6	Current occupation	No job Employed Student Labour worker Retired	79 (32.64) 88 (36.36) 51 (21.07) 21 (8.68) 3 (0.01)	15 24 17 3 1	43 49 27 9 2	8 8 1 5 0	13 7 6 4 0	0.869
7	Monthly income (ETB)	<500 500 – 1000 1001 – 5000 >5000	19 (7.85) 36 (14.88) 119 (49.17) 68 (28.10)	7 4 38 11	7 24 60 39	2 3 7 10	3 5 14 8	0.112

### Clinical characteristics and disease profiles of IBD patients

Among patients with CD, the most common disease location was ileocolonic (51.03%), followed by ileal (32.63%) and colonic involvement (8.95%), with a smaller percentage presenting with perianal disease (7.37%). In terms of disease behavior, most CD cases were stricturing (41.05%) or non-stricturing/non-penetrating (38.42%), while a smaller proportion had penetrating disease (20.53%).

For patients with UC, pancolitis was the most common disease extent (44.23%), followed by proctitis (28.85%) and left-sided disease (26.92%). Nearly all participants were non-smokers (98.76%), and about one-third (31.82%) had undergone surgery related to IBD.

Approximately one-quarter (23.14%) had a history of hospitalization due to IBD, with most of these patients (60.71%) experiencing only a single hospitalization. The vast majority (92.15%) did not report chronic comorbidities; the remaining 7.85% had conditions such as psychiatric disorders, diabetes, hypertension, or stroke.

Regarding IBD treatment, 32 patients (13.22%) were not taking any medication. Among those on treatment, 147 (60.74%) were receiving immunomodulators, 50 (20.66%) were on combination therapy involving more than one medication class, 7 (2.89%) were taking azathioprine or 5-ASA, and 6 (2.48%) were on steroids. No patients were receiving biologic therapy (**Table 2**).

**Table 2:** Clinical characteristics and disease profiles of IBD patients in Addis Ababa, Ethiopia, 2024.

No	Variables	Category	Total	CD		UC		P value
				Active	Non active	Active	Non active	
1	Disease location for (CD)	Ileal Colonic Ileocolonic Perianal	62 (32.63) 17 (8.95) 97 (51.03) 14 (7.37)	25 3 25 7	37 14 72 7			0.033
2	Disease behaviour for (CD)	None of both Stricturing Penetrating	73 (38.42) 78 (41.05) 39 (20.53)	19 26 15	54 52 24			0.367
3	Disease extent for (UC)	Proctitis Pancolitis left-sided	15 (28.85) 23 (44.23) 14 (26.92)			5 10 7	10 13 7	0.655
4	Disease duration at inclusion (years)	Mean ±SD	4.06± 3.73	4.11 ± 3.65		4.019 ± 3.99		0.3884
5	Medication use for IBD at inclusion	None 5-ASA Steroids Immunomodulators Combined	32 (13.22) 7 (2.89) 6 (2.48) 147 (60.74) 50 (20.66)	6 0 1 40 13	16 0 1 95 18	2 4 2 6 8	8 3 2 6 11	0.246
6	Current smoking at inclusion	Yes No	3 (1.24) 239 (98.76)	2 58	1 129	0 22	0 30	0.227
7	Previous surgery for IBD	Yes No	77 (31.82) 165 (68.18)	18 42	55 75	2 20	2 28	0.076
8	Hospitalizations due to IBD	Yes No	56 (23.14) 186 (76.86)	17 43	29 101	6 16	4 26	0.195
9	Number of hospitalizations	One Greater than one	34 (60.71) 22 (39.28)	11 6	19 10	2 4	2 2	0.629
10	Chronic comorbidities	Yes No	19 (7.85) 223 (92.15)	5 55	8 122	2 20	4 26	0.480

### Biochemical and nutritional characteristics of IBD patients

The majority of individuals (81.82%) did not use nutritional supplements or vitamins, while only a small proportion (18.18%) reported usage. Regarding body mass index (BMI), most individuals were classified as normal weight (52.07%), while 32.23% were underweight, 11.98% overweight, and 3.72% obese.

For mid-upper arm circumference (MUAC), the majority (78.10%) were within the normal range, while 15.29% had moderate malnutrition and 6.61% had severe malnutrition. According to the Malnutrition Universal Screening Tool (MUST), 34.30% of patients were at high risk of malnutrition, 21.90% at medium risk, and 43.80% at low risk. Based on the Subjective Global Assessment (SGA), more than half of the patients were moderately (26.86%) or severely (24.79%) malnourished, while 48.35% were considered well-nourished.

In terms of inflammatory markers, most individuals (155; 64.05%) had normal C-reactive protein (CRP) levels, while 35.95% had elevated levels. For erythrocyte sedimentation rate (ESR), a larger proportion (69.12%) showed elevated levels (**Table 3**).

**Table 3:** Biochemical and nutritional characteristics of IBD patients in Addis Ababa, 2024.

No	Variable	Value	Total (%)	CD		UC		P value
				Active	No- active	Active	Non active	
1	Use of nutritional supplements and vitamins	Yes No	44 (18.18) 198 (81.82)	15 45	24 106	4 18	1 29	0.150
2	BMI Kg/m2	Underweight Normal Overweight Obesity	78 (32.23) 126 (52.07) 29 (11.98) 9 (3.72)	28 29 2 1	40 66 19 5	7 11 2 2	3 20 6 1	0.021
3	MUAC	Severe Moderate Normal	16 (6.61) 37 (15.29) 189 (78.10)	6 14 40	9 18 103	1 3 18	0 2 28	0.138
4	MUST Score Category	Low risk Medium risk High risk	106 (43.80) 53 (21.90) 83 (34.30)	14 16 30	69 26 35	5 5 12	18 6 6	0.000
5	SGA Category	Well nourished Moderate Severe	117 (48.35) 65 (26.86) 60 (24.79)	17 21 22	70 29 31	10 7 5	20 8 2	0.009
6	Hemoglobin	Mean ± SD	13.63±3.09	13.52±3.29		14.04±2.21		0.1368
7	Leucocytes	Mean ± SD	6.50± 2.75	6.20±2.41		7.60±3.56		0.7043
8	C-reactive protein	Normal Elevated	155 (64.05) 87 (35.95)	40 20	86 44	8 14	21 9	0.201
9	ESR	Normal Elevated	74 (30.58) 168 (69.042	23 38	37 92	4 18	9 21	0.570

### Factors associated with active disease of inflammatory bowel disease

In the univariate logistic regression analysis, variables that met the inclusion criterion of p ≤0.25 for entry into the multivariable analysis were: sex, place of residence, marital status, income, type of IBD, history of IBD-related surgery, hospitalizations due to IBD, MUST score, SGA score, BMI, hemoglobin, and CRP.

Finally, in the multivariable analysis, income and MUST score remained statistically significant at a p-value < 0.05 after adjusting for covariates. IBD patients with a monthly income between 500–1000 ETB had an approximately 80% lower risk of active disease at inclusion compared to those with an income of less than 500 ETB (Adjusted Odds Ratio [AOR] = 0.20; 95% CI: 0.05–0.79). Patients classified as high risk for malnutrition based on the MUST score had approximately a fourfold increased risk of active disease compared to those at low risk (AOR = 4.30; 95% CI: 1.69–10.91) (**Table 4**).

**Table 4:** Bivariate and multivariate logistic regression of factors associated with active disease among IBD patients, Addis Ababa Ethiopia, 2024.

No	Variable	Category	Total	Active disease		COR(CI)	P value	AOR(CI)	P value
				Yes	No				
1	Sex	Male Female	83 159	34 48	49 111	1.60(0.92,2.79) Ref	0.094*	1.65(0.82,3.32)	0.158
2	Age in years	Mean ±SD	32.27 ± 10.77			1.01(0.98,1.03)	0.394		
3	Marital status	Single Married Widowed Divorced	113 117 4 8	39 36 2 5	74 81 2 3	Ref 0.84(0.48,1.46) 1.89(0.25,13.93) 3.16(0.71,13.93)	Ref 0.081* 0.128* 0.680	Ref 0.74(0.37,1.49) 3.43(0.26,44.88) 2.28(0.41,12.69)	Ref 0.406 0.346 0.345
4	Place of residence	Rural Urban	228 14	75 7	15 7	Ref 0.49(0.16,1.44)	Ref 0.197*	Ref 0.56(0.16,1.96)	0.367
5	Level of education	No formal Grade 1-8 Grade 9-12 College & above	18 30 82 112	7 13 25 37	11 17 57 75	Ref 1.20(0.36,3.95) 0.68(0.23,1.98) 0.77(0.27,2.16)	Ref 0.762 0.490 0.627		
6	Current occupation	No job Employed Student Labour worker Retired	79 88 52 21 3	23 32 19 8 1	56 56 33 13 2	Ref 1.39(0.72,2.66) 1.32(0.62,2.81) 1.49(0.54,4.09) 1.21(0.10,14.09)	Ref 0.322 0.460 0.431 0.875		
7	Income	500 500 – 1000 1001 – 5000 >5000	19 36 119 68	9 7 45 21	10 29 74 47	Ref 0.26(0.07,0.90) 0.67(0.25,1.78) 0.49(0.17,1.40)	Ref 0.035* 0.430 0.186*	Ref 0.20(0.05.0.79) 0.80(0.26,2.51) 0.68(0.20,2.35)	Ref 0.022** 0.715 0.552
8	Age at diagnosis	Mean ±SD	28.01 ±10.83			1.00(0.98,1.03)	0.553		
9	Type of IBD	CD UC	190 52	60 22	130 30	Ref 1.58 (0.84,2.94)	0.149*	Ref 1.74(0.78,3.85)	0.170
10	Disease duration	Mean ±SD	4.06± 3.73			0.99(0.92,1.06)	0.776		
11	Current smoking	No Yes	239 3	80 2	159 1	Ref 3.97(0.3,44.49)	0.263		
12	Medication use for IBD	None Yes	25 217	5 77	20 140	Ref 1.68(0.69,3.81)	Ref 0.258		
13	History of surgery for IBD	No Yes	165 77	62 20	103 57	Ref 0.58(0.321.06)	0.077*	0.69(0.33,1.46)	0.344
14	Hospitalizations due to IBD	No Yes	186 56	59 23	127 33	Ref 1.50(0.81,2.77)	0.196*	1.70(0.80,3.58)	0.161
15	Chronic comorbidities	No Yes	223 19	122 9	101 10	Ref 1.15(0.43,3.04)	0.77		
16	Haemoglobin	Mean ± SD	13.63±3.09			0.91(.80, 1.02)	0.129*	0.93(0.79,1.10)	0.456
17	Leucocytes	Mean ± SD	6.50± 2.75			0.98(.88 ,1.08)	0.703		
18	Must score	Low Medium High	106 53 83	10 26 46	79 32 39	Ref 3.00(1.43,6.30) 4.69(2.43,9.04)	Ref 0.000* 0.000*	Ref 2.51(0.99,6.37) 4.30(1.69,10.91)	Ref 0.051 0.002**
19	SGA score	Grade A Grade B Grade C	117 65 60	27 28 27	86 37 37	Ref 2.52(1.25,4.63) 2.72(1.20,4.48)	Ref 0.005* 0.003*	Ref 1.16(0.47,2.90) 0.93(0.27,3.21)	Ref 0.737 0.919
20	CRP	Normal Elevated	198 44	65 17	133 27	Ref 1.04(0.59,1.83)	Ref 0.873		
21	BMI	Underweight Normal Overweight Obesity	78 126 29 9	35 40 4 3	43 86 25 7	Ref 0.57(0.30,1.01) 0.19(0.059,0.60) 0.61(0.13,2.56)	Ref 0.060* 0.005* 0.512	Ref 0.93(0.37,2.35) 0.46(0.09,2.32) 1.08(0.14,7.89)	Ref 0.895 0.353 0.936

*stands for p-value ≤ 0.25 in bivariate analysis, ** stands for p-value <0.05 in multivariable analysis

## Discussion

Nutritional abnormalities are commonly reported in patients with inflammatory bowel disease (IBD), particularly among those with active disease (41, 42). While nutritional assessments such as body mass index (BMI) and vitamin levels have been studied in IBD patients (24, 43), the role of other nutritional assessment tools—such as the Subjective Global Assessment (SGA) and the Malnutrition Universal Screening Tool (MUST)—has been poorly investigated in Sub-Saharan Africa, including Ethiopia. Therefore, in this study, we aimed to characterize the nutritional profile of IBD patients and examine its relationship with various sociodemographic and clinical factors that may influence disease activity.

We found that 82 (33.88%) of IBD patients had active disease at the time of inclusion. This finding is higher than that reported in other studies (29, 44). However, it is lower than findings reported in other studies (20, 21). This discrepancy might be due to differences in sample size, disease duration, and diagnostic criteria used.

Our findings highlight the high prevalence of malnutrition and its associated risk among this cohort of IBD patients. More than half (54.2%) were found to be at moderate or high risk of malnutrition based on the MUST score. Additionally, MUAC measurements classified approximately 21.9% of patients as malnourished. Early detection of patients at risk of developing malnutrition is critical due to its high prevalence and associated health complications (45). In our study, malnutrition was present in 51.65% of patients based on the SGA category and in 32.23% based on BMI. These rates are consistent with a previous study conducted in China, which reported a prevalence of 49.5% (22). However, our findings exceed those reported in studies conducted in the USA (7.8%) (29), Spain (16%) (46), Turkey (9.9%) (47), and Romania 36.3% (48). On the other hand, some studies have reported an even higher burden of malnutrition, such as those conducted in China 59% (49) and India 52.6% (35). Differences in malnutrition prevalence across studies can be attributed to several factors, including variations in study populations (e.g., patients with active disease, those in remission, newly diagnosed individuals, or hospitalized patients), differences in sample sizes, and the use of differing diagnostic criteria.

The main mechanisms contributing to the high burden of malnutrition in IBD include reduced oral intake, malabsorption, increased gastrointestinal nutrient losses, drug–nutrient interactions, elevated nutrient requirements, increased lipid oxidation, decreased glucose oxidation, reduced diet-induced thermogenesis, and increased resting energy expenditure (50–52).

We found that the MUST score had a significant association with active disease at inclusion in the multivariable analysis after adjusting for covariates. This finding is supported by other studies conducted worldwide (31, 53–56). These studies have shown that patients with active disease are more likely to be malnourished than those with quiescent disease (57). This may be due to the fact that more severe inflammation affects the bowel, leading to reduced absorption of both macro- and micronutrients (54).

In our study, income was also found to be a significant predictor of active disease at inclusion. To our knowledge, this is the first study to demonstrate a direct association between income and clinical disease activity in the context of IBD. However, this finding aligns with previous reports indicating that individuals with higher income and better socioeconomic status are at a decreased risk of experiencing active disease.

The role of income as a predictor of active disease in IBD—including Crohn’s disease and ulcerative colitis—is consistent with findings from earlier studies (58). Research has demonstrated that socioeconomic status, often measured by income, plays a crucial role in the onset and progression of IBD. Lower income levels have been associated with an increased risk of developing IBD and with more severe disease activity (59).

This may be attributed to several factors, including reduced access to healthcare, lower dietary quality, higher stress levels, and poorer living conditions—all of which can exacerbate inflammatory responses in the body (60, 61). For instance, research by Sun et al. (62) highlighted the influence of lower socioeconomic status, concluding that lower income correlates with a higher IBD symptom burden and reduced social participation, both of which can worsen health conditions (62).

Additionally, in the univariate analysis, factors such as SGA category, BMI, and history of IBD treatment exposure were found to be significant predictors of clinical disease activity. However, in the multivariable analysis, these variables lost statistical significance after adjusting for covariates. One possible explanation is that these factors may have confounding relationships with stronger predictors of disease activity, thereby diminishing their independent effect in the multivariable model. Moreover, interactions among various predictors may dilute the individual influence of some variables observed in the univariate analysis, rendering them non-significant in the adjusted model.

Although these variables were not statistically significant in the multivariable analysis, their potential clinical relevance should not be overlooked.

### Strength and limitations

To our knowledge, this is the first study in this setting—and in Ethiopia—to assess the effects of nutritional factors on disease activity among patients with IBD. The study was conducted in two different healthcare facilities, which enhances the generalizability of the findings.

However, the study has some notable limitations. Due to its cross-sectional design, it is not possible to establish a temporal relationship between nutritional abnormalities and disease activity in IBD patients. Although multiple nutritional indicators were used, reliance on SGA, BMI, and MUAC may not fully capture the complexity of malnutrition in this population. The absence of additional measures—such as dietary intake or micronutrient levels—which could offer a more comprehensive assessment, is another limitation. Furthermore, the study is subject to potential selection bias, as it was conducted exclusively in healthcare settings.

## Conclusion and recommendations

Active disease at inclusion was observed in approximately one-third of patients with IBD. Severe malnutrition and low-income status were significantly associated with clinically active IBD. Early and comprehensive nutritional assessment at diagnosis, as well as periodic reassessment during follow-up, is strongly recommended. Nutritional management should be prioritized for patients identified with malnutrition in routine clinical practice.

Future research should explore the impact of early nutritional interventions on IBD disease activity and clinical outcomes, including hospitalization and mortality.

The public health implications of these findings underscore the urgent need for initiatives that address both nutritional deficiencies and socioeconomic disparities among patients with IBD. Clinically, the study emphasizes the importance of routine nutritional assessments and consideration of socioeconomic factors in IBD management. Tailored interventions based on these assessments may lead to more effective disease control and improved overall patient outcomes.

## Data Availability

The raw data supporting the conclusions of this article will be made available by the authors, without undue reservation.
